# Diabetes Mellitus-Related Neurobehavioral Deficits in Mice Are Associated With Oligodendrocyte Precursor Cell Dysfunction

**DOI:** 10.3389/fnagi.2022.846739

**Published:** 2022-05-25

**Authors:** Li-Ping Wang, Jieli Geng, Chang Liu, Yuyang Wang, Zhijun Zhang, Guo-Yuan Yang

**Affiliations:** ^1^Department of Neurology, Renji Hospital, Shanghai Jiao Tong University School of Medicine, Shanghai, China; ^2^Med-X Research Institute and School of Biomedical Engineering, Shanghai Jiao Tong University, Shanghai, China; ^3^Department of Rehabilitation Medicine, The Affiliated Hospital of Qingdao University, Qingdao University, Qingdao, China

**Keywords:** cognition, behavior, diabetes, oligodendrocyte precursor cells, white matter

## Abstract

Recent clinical studies demonstrated an increase of the incidence of neurobehavioral disorders in patients with diabetes mellitus. Studies also found an association between severity of diabetes mellitus and the progression of white matter hyperintensity on magnetic resonance imaging, which conferred risk for developing cognitive impairment. Since oligodendrocyte precursor cells participated in the white matter repair and remodeling after ischemic brain injury, we explored whether hyperglycemia induced neurobehavioral deficits were associated with dysfunction of oligodendrocyte precursor cells. Adult male C57BL/6 mice (*n* = 40) were randomly divided into 4-week diabetes, 8-week diabetes, and control groups. Experimental diabetic mice were induced by streptozotocin injection. Learning and cognitive function, exploratory, anxiety and depression behaviors were assessed by Morris water maze, open field test, elevated plus maze, and tail suspension test, respectively. Immunofluorescence staining of neuron-glial antigen 2 and myelin basic protein were performed. Oligodendrocyte precursor cells were cultured in different glucose level to explore possible mechanism *in vitro*. The learning and cognitive function of 4-week and 8-week diabetic mice were attenuated compared to the control group (*p* < 0.05). The diabetic mice had less exploratory behavior compared to the control (*p* < 0.05). However, the diabetic mice were more likely to show anxiety (*p* < 0.05) and depression (*p* < 0.01) compared to the control. Further study demonstrated the number of oligodendrocyte precursor cells and the level of myelin basic protein expression were decreased in diabetic mice and the migration and survival ability were suppressed in the hyperglycemic environment *in vitro* (*p* < 0.05). Our results demonstrated that diabetes mellitus induced neurological deficits were associated with the decreased number and dysfunction of oligodendrocyte precursor cells.

## Introduction

Diabetes mellitus is one of the largest global public health concerns, imposing a heavy global burden on public health (Lin et al., 2020). The overall prevalence is 9.1% and increases rapidly with the age in China. The prevalence of the 65–74 years group is as high as 14.1% (Yang et al., 2016). The quantitative meta-analysis indicates that diabetes mellitus is a risk factor for incident dementia and subjects with diabetes mellitus have an increased risk of developing dementia (Cheng et al., 2012). The prevalence of anxiety and depression symptoms in patients with diabetes mellitus is considerably higher than that in general population samples (Collins et al., 2009).

Cognitive dysfunction and emotional disorders are closely associated with destruction of white matter connectivity. The white matter hyperintensity seen on magnetic resonance imaging confers risk for developing cognitive impairment (King et al., 2014). About 2 longitudinal studies found a significant association between diabetes mellitus and the progression of white matter hyperintensity. Also 1 study followed 113 subjects aged over 60 years for 2 years, and concluded that patients with diabetes mellitus showed a larger volume and increasing rate of white matter hyperintensity. Another study followed 396 elderly subjects for 3 years, and found that diabetes mellitus affected the progression of the white matter hyperintensity (Tamura and Araki, 2015).

The mechanism of brain injury with hyperglycemia is complicated. Diabetic vasculopathy or angiopathy was long considered as a cause of brain injury with chronic hyperglycemia. Experimental and clinical studies showed that hyperglycemia resulted in increased neuronal and astrocytic oxidative stress injuries, necrotic brain damage and neuroinflammation, apoptotic neuronal death, impairment of brain repair processes, and suppression of neuronal cell proliferation. As previous study reported, diabetes mellitus could cause white matter damage (Zhang et al., 2016; Ma et al., 2018). White matter consists of axons, oligodendrocytes, and astrocytes. Oligodendrocytes are the myelinating cells responsible for axonal myelination and are highly susceptible to damages. Oligodendrocyte precursor cells (OPCs) mediate the remyelination process after white matter injury and play an important role in the repair of central nervous system injury (Wang et al., 2016). However, whether the function of OPCs in hyperglycemic environment is affected and then alleviates the white matter repair is still unknown.

In present study, we use streptozotocin (STZ) induced diabetic mouse model to explore whether hyperglycemia induced neurobehavioral deficits are associated with OPC dysfunction.

## Materials and Methods

### Experimental Design

**FIGURE 1:**
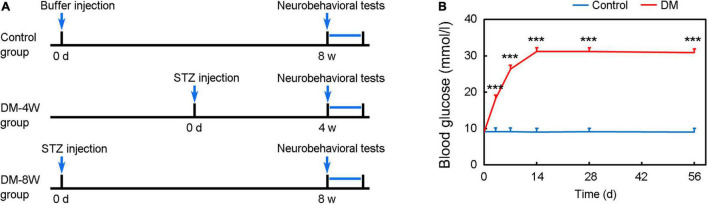
Experimental design and establishment of a hyperglycemic mouse model. **(A)** Diagram of experimental design. **(B)** Establishment of hyperglycemic mouse model. Line chart shows the blood glucose level after STZ injection. Data are mean ± SD, *n* = 12 per group, ***p < 0.001.

Animal experimental procedures were approved by the Institutional Animal Care and Use Committee (IACUC) of Shanghai Jiao Tong University, Shanghai, China. Animal studies were reported according to ARRIVE guidelines. Adult male C57BL/6mice (*n* = 40) weighing 26–28 g (Jeisijie, Shanghai, China) were randomly divided into 3 groups: normal group (as a control), 4-week diabetes group (DM-4W), and 8-week diabetes group (DM-8W), *n* = 12 per group (Figure 1A). The duration of diabetes is the key factor for the incidence of dementia (Ryan et al., 2003). As we wanted to explore whether the duration of diabetes could affect the neurobehavioral deficits, we designed two DM groups. Animals were housed with free access to water and food.

### Induction of Diabetes Mellitus in Mice

Diabetes was induced by a single injection of STZ (150 mg/kg, Sigma-Aldrich, St. Louis, MO, United States) (Geng et al., 2017). STZ was freshly dissolved in 0.1 M sodium citrate buffer (pH 4.5) to a final concentration of 10 mg/ml and used within 20 min. Before STZ administration, mice were fasted (water was available) for 10 h. After STZ injection, mice were allowed access to food and water freely. At 3 days after STZ injection, diabetes status was assessed by measuring serum glucose levels using the glucose oxidase method (Bayer HealthCare LLc, Mishawaka, IN, United States). Diabetes is defined as serum glucose concentration being above 300 mg/dl as our previously reported (Mu et al., 2016; Geng et al., 2018).

### Learning and Cognition-Morris Water Maze

The Morris water maze test was performed as described on the nature protocols (Vorhees and Williams, 2006). All trials were performed in a quiet room with indirect lighting. The apparatus was acircular tank with 122 cm in diameter as a swimming pool, and contained water at approximately 20–22°C. The swimming pool was virtually divided into 4 equal quadrants. The edible pigment was used to opaque water that helps to camouflage the submerged platform. The hidden circular platform of 10 cm^2^ in size located in the quadrant of northeast (NE) and submerged 1.0 cm below the water surface and remained constant during the entire spatial acquisition trials.

### Exploration-Open Field

The open field test is used to assess anxiety and exploratory behavior (Seibenhener and Wooten, 2015). Normal animals explore or hide to protect themselves. So they spend more time on the periphery of the open field area than in the center (the most anxiogenic area). In the open field (40 × 40 × 30 cm), the peripheral area and the central area each account for 50% of the area. The mice were placed in the center of the open-field apparatus, and the video tracking system was used to record the time, distance and number of crossings of the mice in the central area within 10 min.

### Anxiety-Elevated Plus Maze

The elevated plus maze testis used to evaluate anxiety-related behaviors in rodent models of central nervous system diseases (Komada et al., 2008). The elevated plus maze equipment consists of a “+”-shaped maze elevated above the ground (75 cm) with 2 opposite closed arms, 2 opposite open arms, and a central area (Figure 4A). This model is based on the test animals’ aversion to open spaces when feeling anxious. As the test mice freely explored the maze, their behavior was recorded by a video camera installed above the maze. Choice behavior was observed for 10 min and the time, distance and number of times the mice entered the open arms were counted.

### Depression-Tail Suspension Test

The tail suspension test is a test for the antidepressant activity of mice (Cryan et al., 2005). In the test, the rear 1/3 of the tail of the mouse is suspended on a lever with tape, and the camera is used within 8 min to record its behavior. In the test, mice struggle to escape for a period of time and then adopt a posture of immobility. Depression decreases the frequency and duration of locomotor activity in mice. After 2 min of induction time, the time for each mouse to keep immobility and the total time are recorded within left 4 min.

### Immunofluorescence Staining

As previously described (Wang et al., 2020), brain slices were fixed with 4% paraformaldehyde and blocked with 10% bovine serum albumin (BSA) (Sigma), and then incubated with primary antibodies of myelin basic protein (MBP, 1:200, Abcam, MA) and neuron-glial antigen 2 (NG2, 1:200, Millipore, Bedford, MA) at 4°C overnight. After rinsing three times with PBS, brain sections were further incubated with second antibodies at room temperature for 1 h. Brain sections were photographed using a confocal microscope (Leica, Solms, Germany). For the quantification of MBP, 2 fields in the striatum area for each slice were randomly collected and analyzed with Image *J* software (National Institutes of Health, Bethesda, MD) for mean integrated density analysis. We calculated the number of NG2^+^ cell near the subventricular zone, and analyzed the number of MBP^+^ white matter fiber and fluorescence area of MBP in the striatum area.

### Western Blot Analysis

Western blot was performed as described previously (Wang et al., 2021). Briefly, the membranes were incubated with primary antibodies against MBP (1:1000, Abcam), and β-actin (1:1000, Abcam) overnight at 4°C. After washing with TBST buffer, the membranes were incubated with HRP-conjugated secondary antibody.

### Oligodendrocyte Precursor Cell Isolation and Identification

The brain cortex from P1 Sprague-Dawley rat pups was dissociated into a single-cell suspension by trypsinize at 37°C for 10 min and the suspension was filtered with a 70-um filter. The cells were seeded on poly-d-lysine (PDL, Sigma-Aldrich, San Louis, MO) coated culture flasks in DMEM (Corning, New York, NY) with 10% fatal calf serum (Gibco, Carlsbad, CA). After 8 days, the microglia was separated from glia cell mixtures after 30 min of culture by a 220 rpm shake. Then cells were collected by 20 h of culture by a 200 rpm shake to inject into the mice or seed on a PDL coated culture dish in Neurobasal-A (Gibco) containing 2% B27 (Gibco), 10 ng/ml PDGF-AA (Gibco), 10 ng/ml bFGF (Peprotech, NJ, United States) and 2 mmol/l glutamine (Gibco) (Yuan et al., 2018; Wang et al., 2020).

Cells were fixed with 4% paraformaldehyde for 5 min at room temperature and blocked by 10% bovine serum albumin. Then OPCs were incubated with primary antibodies against PDGFR-α (1:100, Santa Cruz Biotechnology, Santa Cruz, CA) and NG2 (1:200, Millipore) at 4°C overnight. Cells were incubated with the fluorescence conjugated second antibodies for 1 h at room temperature.

### Oligodendrocyte Precursor Cell Migration Assay

Migration assay was performed as previously described (Miyamoto et al., 2008). Briefly, the migration test was performed in 24 mm transwell with 8.0 μm pore polycarbonate membrane inserts (Corning). The transwell chambers were coated with PDL (Sigma-Aldrich, San Louis, MO) for 12 h. 2 × 10^5^ OPCs suspended in growth factors-free Neurobasal-A with 6, 25 or 50 mmol/L glucose were added to the upper chamber. After 18 h, chambers were stained with crystal violet (Beyotime) for 5 min. The number of OPCs that migrated to the lower membrane surface was counted manually in 5 random fields, and 4 chambers were counted for each group.

### Statistical Analysis

Samples size was estimated using a type I error rate of 0.05 and a power of 0.8 on a 2-sided test by power analysis. Analysis was performed by SPSS. Multiple comparisons were analyzed using one-way ANOVA followed by Tukey’s *post hoc* test. Data were expressed as mean ± SD. A probability value less than 0.05 were considered significant.

## Results

### The Establishment of a Hyperglycemic Mouse Model

Blood glucose significantly increased after STZ administration compared to control and maintained at a high level until 8 weeks (Figure 1B, *p* < 0.001).

### Learning and Cognition Behavior Attenuated in Diabetic Mice

**FIGURE 2:**
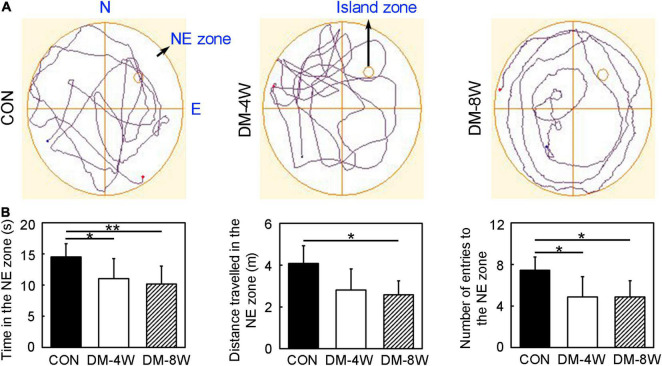
Learning and cognition behavior attenuated in diabetic mice. **(A)** The track plot showed the mouse trace of the control, DM-4W and DM-8W groups in the probe test. **(B)** Bar graphs showed the time in the NE zone, the distance traveled in the NE zone and number of entries to the NE zone. The island zone was located in the NE zone. Data are mean ± *SD*, *n* = 8 per group, *p < 0.05, DM-4W or DM-8W vs. control; **p < 0.01, DM-8W vs. control.

We performed Morris water maze to detect the learning and cognition (Figure 2A). The time in the NE zone, the distance traveled in the NE zone and number of entries to the NE zone in the probe test of DM-4W and DM-8W groups were all decreased compared to the control (Figure 2B, *p* < 0.05), which indicating the learning and cognition deficits of diabetic mice. However, there was no significant difference between group DM-4W and DM-8W in these indicators.

### Anxiety and Exploratory Behavioral Changes in Diabetic Mice

**FIGURE 3:**
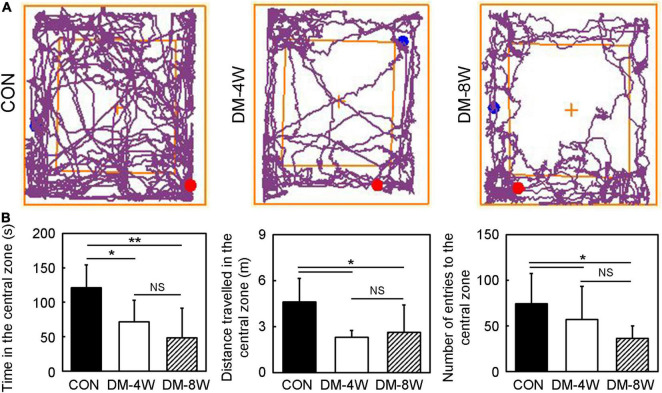
Anxiety and exploratory behavior increased in diabetic mice. **(A)** The track plot showed the mouse trace of the control, DM-4W and DM-8W groups in the open field test. **(B)** Bar graphs showed the time in the central zone, the distance traveled in the central zone and number of entries to the central zone. Data are mean ± *SD*, *n* = 8 per group, *p < 0.05, DM-4W or DM-8W vs. control;**p < 0.01, DM-8W vs. control.

We performed open field test to estimate the anxiety and exploratory behavior of mice (Figure 3A). The time in the central zone, the distance traveled in the central zone and the number of entries to the central zone of DM-4W and DM-8W groups were all decreased compared to the control, but no difference between the DM-4W and DM-8W mice (Figure 3B, *p* < 0.05), indicating that diabetic mice exhibit more anxious behavior than normal mice.

**FIGURE 4:**
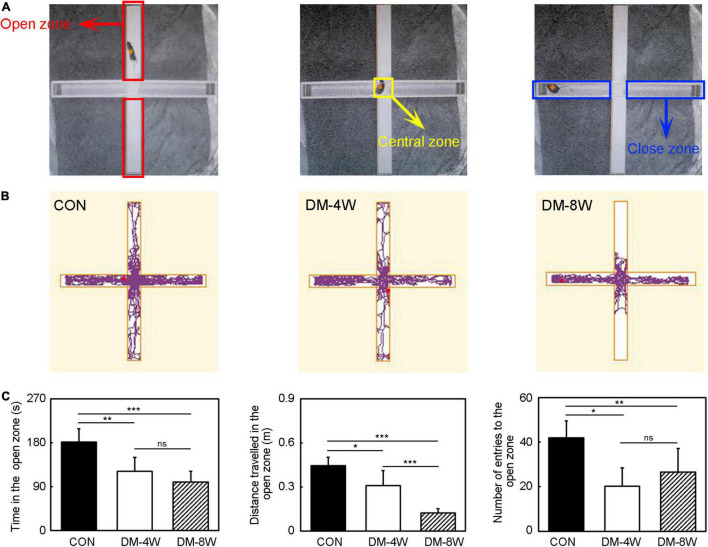
Anxiety behavior increased in diabetic mice. **(A)** The diagram of the elevated plus maze zone. The red box indicates the open zone. The yellow box indicates the central zone. The blue box indicates the close zone. **(B)** The track plot showed the mouse trace of the control, DM-4W and DM-8W groups in the elevated plus maze. **(C)** Bar graphs showed the time in the open zone, the distance traveled in the open zone and number of entries to the open zone. Data are mean ± *SD*, *n* = 8 per group, *p < 0.05, **p < 0.01, ***p < 0.001.

### Anxiety Behavior Increased in Diabetic Mice

We performed elevated plus maze test to observe anxiety behavioral (Figure 4B). In the elevated plus maze test, the time in the open zone, the distance traveled in the open zone and the number of entries to the open zone of DM-4W and DM-8W groups were all reduced compared to the control (Figure 4C, *p* < 0.05), indicating that diabetic mice feel more anxious than normal mice. There were no significant differences between DM-4W and DM-8W mice of the time in the open zone and the number of entries to the open zone. However, DM-8W mice presented a significant decrease in the distance traveled in the open zone compared to DM-4W mice (Figure 4C, *p* < 0.001).

### Depression Behavior Increased in Diabetic Mice

**FIGURE 5:**
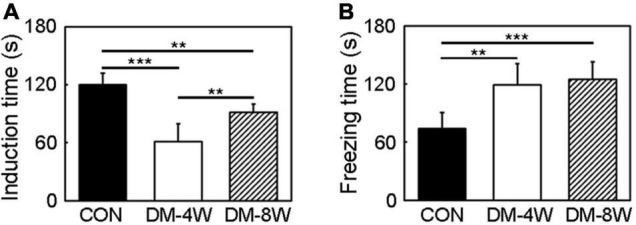
Depression behavior increased indiabetic mice. **(A)** Bar graph of induction time of the control, DM-4W and DM-8W groupsin the tail suspension test. **(B)** Bar graph of freezing time of the control, DM-4W and DM-8W groups in the tail suspension test. Data are mean ± *SD, n* = 8 per group, **p < 0.01, ***p < 0.001.

The depression-like behavior was assessed by tail suspension test. The induction time indicated depression level. The increase of the induction time showed that animals tended to be more depressed. The induction time significantly decreased in diabetic mice compared to the control especially in DM-4W mice (Figure 5A, *p* < 0.01), meanwhile the freezing time significantly increased in diabetic mice (Figure 5B, *p* < 0.01).

### The Number of Oligodendrocyte Precursor Cell and Myelin Basic Protein Expression Were Decreased in Diabetic Mice

**FIGURE 6:**
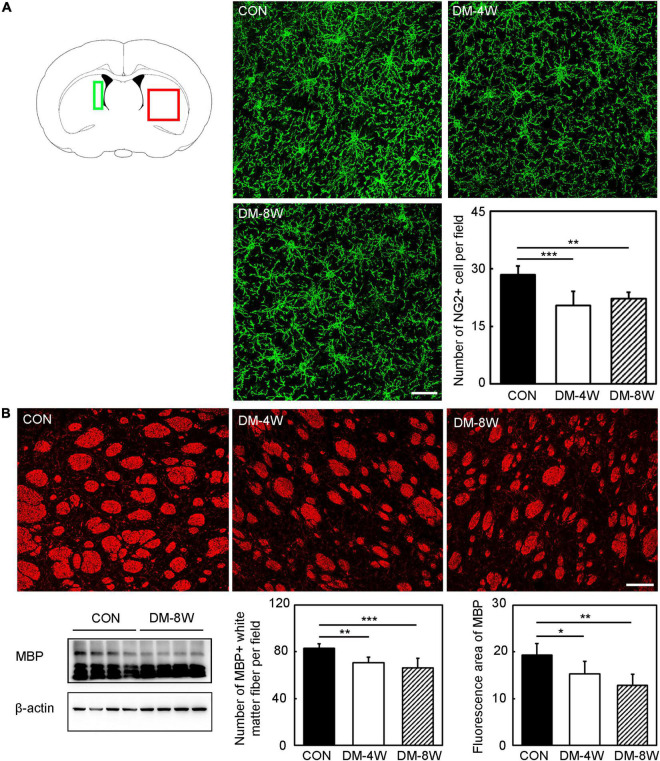
The number of OPC and MBP expression decreased in diabetic mice**. (A)** Immunofluorescent images of NG2 (green) and bar graph of NG2 quantification in the control, DM-4W and DM-8W groups. Scale bar = 50 μm. The green box indicates the location of the NG2 immunofluorescence staining pictures collected by confocal microscope. The red box indicates the location of the MBP immunofluorescence staining pictures collected by confocal microscope. **(B)** Immunofluorescent and Western blot images of MBP and bar graphs of MBP quantification in the control, DM-4W and DM-8W groups. Scale bar = 100 μm. Data are mean ± *SD*, *n* = 6 per group, *p < 0.05, **p < 0.01, ***p < 0.001, DM-4W or DM-8W vs. control.

To test whether diabetes mellitus could affect the number of OPC and MBP expression, brain sections were stained with NG2 and MBP. We found that the number of OPC (Figure 6A, *p* < 0.01) and the expression of MBP in white matter fiber (Figure 6B, *p* < 0.05) were reduced in diabetic mice.

### Hyperglycemia Eliminated the Oligodendrocyte Precursor Cell Migration and Survival Ability

**FIGURE 7:**
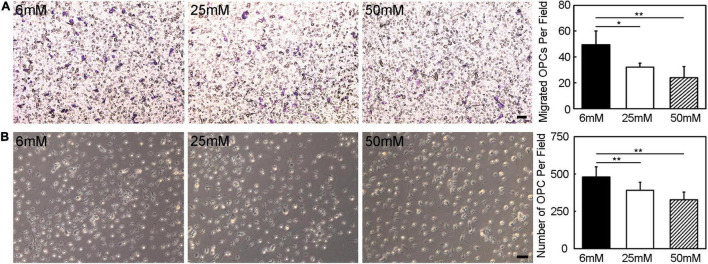
Hyperglycemia eliminated the OPC migration and survival ability. **(A)** The migration ability of OPCs was assessed by transwell *in vitro*. Images showed the migrated OPCs in the 6 mM, 25 mM, and 50 mM glucose in 18 h cultured condition. Scale bar = 50 μm. **(B)** Images and bar graph showed the number of survived OPCs in the 6 mM, 25 mM and 50 mM glucose in 3-day cultured condition. Scale bar = 50 μm. Data are mean ± *SD*, *n* = 6 per group, *p < 0.05, **p < 0.01, 25 mM or 50 mM vs. 6 mM.

In migration assay, the migrated cell number decreased obviously with the increase of glucose concentration, which was reduced by half under 50 mmol/L glucose compared to 6 mmol/L glucose (Figure 7A, *p* < 0.05). The number of OPCs also decreased with the increase of glucose concentration (Figure 7B, p < 0.01).

## Discussion

Previous studies paid more attention to the physical symptoms of diabetes mellitus and complications including vision problems, foot complications, high risk of wound infection, and etc. (Zhang et al., 2010; Forsythe et al., 2015; Martin et al., 2016). However, diabetes mellitus impacts cognitive and mental health (Dolan et al., 2018).

In children with type 1 diabetes mellitus showed poor sustained and divided attention, impairment of new learning, mental efficiency, low receptive language scores, low intelligence quotient (IQ), and poor academic achievement. In adults with type 1 diabetes mellitus displayed deficits in psychomotor efficiency and motor speed, vocabulary and verbal IQ, information processing speed, mental flexibility, visual perception, visual and sustained attention, episodic memory, working memory, visual memory, executive function, and low IQ. In adults with type 2 diabetes mellitus exhibited deficits in psychomotor speed, processing speed, complex motor functioning, verbal memory, working memory, immediate recall, delayed recall, verbal fluency, visual retention, attention; frontal lobe/executive function, semantic categorization, and overall cognition (Kim, 2019). In this study, we established type 1 diabetes mellitus model and performed Morris water maze to detect the new learning and memory of diabetic mice. Our result showed that persistent hyperglycemia impaired the new learning and memory ability which was consistent with previous clinical studies.

To explore whether diabetes mellitus could affect the exploratory behaviors, we used open field test to evaluate the neurobehavior of diabetic mice. The open field test is based on the natural tendency of an animal to explore and to protect itself using avoidance which translates to a normal animal spending more time in the periphery than in the center (Seibenhener and Wooten, 2015). So, this task could explore the exploratory drive. Interestingly, diabetic mice more intended to stay in the peripheral zone close to the walls of experimental box, and had less courage to step into the central zone. Previous study also reported that db/db mice underwent a decrease in basic movements and fine movements compared to age-matched controls in the open field test (Sharma et al., 2010).

Our study assessed the anxiety of type 1 diabetic mice by open field test and elevated plus maze. Both tests revealed that diabetic mice were more anxious than control mice.

Furthermore, when compared to the open field test, the elevated plus maze appeared to be more sensitive to detecting diabetic mice anxiety. Contrary to a previous study about type 2 diabetes mellitus, db/db mice were observed to be less anxious than age-matched lean controls in the elevated plus maze test. But the spontaneously diabetic INS2^Akita^ mouse was reported anxiety-like behavior using the elevated plus maze test (Asakawa et al., 2007). Clinical studies evaluating anxiety levels in type-2 diabetic patients also led to conflicting outcomes.

Patients living with type 1 or type 2 diabetes are at increased risk for depression. Rates of depression across the lifespan are 2 times greater for patients with diabetes than in the general population (Ducat et al., 2014). The tail suspension test indicated that diabetic mice tended to be more depressed than control. Our study was consistent with clinical studies. Because the diabetic mice could not endure the long-time swimming, we did not choose the forced swimming test to assess depression. The tail suspension test was safer for diabetic mice.

The worse neurological deficits in patients with diabetes may correlate with the disruption of white matter connections. To date, the majority of the studies on white matter injury of diabetes mellitus are clinical imaging studies. Few preclinical studies on the relationship between white matter injury and cognitive impairment mainly focused on diabetic animals. Our study proved diabetes mellitus caused the myelin damage. An important finding is that the number of OPCs is decreased. Further study *in vitro* proved the finding hyperglycemic culture affect the survival ability of OPCs. Besides, the migration ability of OPCs was weakened in hyperglycemic culture. OPCs, which are widely distributed throughout the adult CNS can differentiate into mature oligodendrocytes covering the axons to form myelin sheath (Wang et al., 2016). Therefore, OPCs could mediate the remyelination process after brain injury. Adult OPCs migrate to the injury site following acute demyelination in the central nervous system (Skaper, 2019). Only a small amount of OPCs is capable to migrate to the ischemic region and differentiate into mature myelin after ischemic stroke (Wang et al., 2021). As the number and function of OPCs were affected by hyperglycemic environment, the repair progress of white matter injury hindered. Our finding consisted with a previous study. The study found that the type 2 diabetes mellitus mice decreased proliferation and survival of OPCs could participate in the progression of white matter lesions after chronic ischemia (Yatomi et al., 2015). This finding might explain why the white matter injury caused by diabetes mellitus could not be repaired by OPCs and resulted in the development of diabetes-associated neurological impairment.

The subventricular zone plays a role in adult neurogenesis, particularly following injury and disease. OPCs could migrate from the subventricular zone to the subcortical white matter and other brain regions (Adams et al., 2020). So, we calculated the number of NG2+ cell near the subventricular zone. The MBP immunofluorescence staining referred to a previous study which quantified the myelin fibers in the striatum (Zhang et al., 2020). The MBP immunofluorescence staining in cortex presented linear appearance instead of fibers. So, we analyzed the number of MBP+ white matter fiber and fluorescence area of MBP in the striatum area.

Previous study reported that diabetes mellitus impaired oligodendrogenesis after stroke. The diabetic mice were significantly less active than WT mice and traveled shorter total distances in the area in the open field test which suggested a reduction in overall exploratory/locomotor activity in diabetic mice (Ma et al., 2018). And OPC dysfunction was a candidate pathophysiological mechanism of familial schizophrenia (de Vrij et al., 2019). Transplantation of OPCs improved the cognitive dysfunction in Morris water maze test in an experimental model of neonatal periventricular leukomalacia (Kim et al., 2018).

In present study, we demonstrated that diabetes mellitus induced neurological deficits included learning and memory deficit, exploratory ability decline, anxiety, and depression. Our study also found the neurological deficits was associated with the decreased number and dysfunction of OPCs. Our study will help to find the therapeutic target for diabetes mellitus related neurobehavioral deficits.

## Data Availability Statement

The raw data supporting the conclusions of this article will be made available by the authors, without undue reservation.

## Ethics Statement

The animal study was reviewed and approved by Institutional Animal Care and Use Committee (IACUC) of Shanghai Jiao Tong University, Shanghai, China.

## Author Contributions

L-PW, JG, and G-YY contributed to conception and design of the study. L-PW and CL collected the data and wrote the first draft of the manuscript. YW performed the statistical analysis. JG, ZZ, and G-YY wrote sections of the manuscript. All authors contributed to manuscript revision, read, and approved the submitted version.

## Conflict of Interest

The authors declare that the research was conducted in the absence of any commercial or financial relationships that could be construed as a potential conflict of interest.

## Publisher’s Note

All claims expressed in this article are solely those of the authors and do not necessarily represent those of their affiliated organizations, or those of the publisher, the editors and the reviewers. Any product that may be evaluated in this article, or claim that may be made by its manufacturer, is not guaranteed or endorsed by the publisher.
